# A Novel Technique for Treatment of Metaphyseal Voids in Proximal Humerus Fractures in Elderly Patients

**DOI:** 10.3390/medicina58101424

**Published:** 2022-10-10

**Authors:** Stoyan Hristov, Luke Visscher, Jörg Winkler, Daniel Zhelev, Stoyan Ivanov, Deyan Veselinov, Asen Baltov, Peter Varga, Till Berk, Karl Stoffel, Franz Kralinger, Boyko Gueorguiev

**Affiliations:** 1 Department of Orthopedics and Traumatology, University Hospital for Active Treatment, 8018 Burgas, Bulgaria; 2 AO Research Institute Davos, 7270 Davos, Switzerland; 3 School of Medicine, Queensland University of Technology, Brisbane 4000, Australia; 4 Cantonal Hospital Graubuenden, 7000 Chur, Switzerland; 5 Department of Orthopaedics and Traumatology, Medical University of Varna, 9002 Varna, Bulgaria; 6 Bulgarian Academy of Sciences, Institute of Metal Science ‘Acad. A. Balevski’, 1574 Sofia, Bulgaria; 7 Department of Trauma Surgery, University Multiprofile Hospital for Active Treatment and Emergency Medicine ‘N. I. Pirogov’, 1606 Sofia, Bulgaria; 8 Department of Traumatology, University Hospital Zurich, 8091 Zurich, Switzerland; 9 Department of Orthopaedics and Traumatology, University Hospital Basel, 4031 Basel, Switzerland; 10 Department of Trauma Surgery, Medical University of Vienna, 1090 Vienna, Austria; 11 Trauma and Sports Department, Teaching Hospital Medical University of Vienna, Ottakring Clinic, 1160 Vienna, Austria

**Keywords:** augmentation, bone cement, defect, fracture, proximal humerus

## Abstract

*Background and Objectives*: The treatment of proximal humerus fractures in elderly patients is challenging, with reported high complication rates mostly related to implant failure involving screw cut-out and penetration. Metaphyseal defects are common in osteoporotic bone and weaken the osteosynthesis construct. A novel technique for augmentation with polymethylmethacrylate (PMMA) bone cement was developed for the treatment of patients in advanced age with complex proximal humerus fractures and metaphyseal voids, whereby the cement was allowed to partially cure for 5–7 min after mixing to achieve medium viscosity, and then it was manually placed into the defect through the traumatic lateral window with a volume of 4–6 mL per patient. The aim of this retrospective clinical study was to assess this technique versus autologous bone graft augmentation and no augmentation. *Materials and Methods*: The outcomes of 120 patients with plated Neer three- and four-part fractures, assigned to groups of 63 cases with no augmentation, 28 with bone graft augmentation and 29 with cement augmentation, were assessed in this study. DASH, CS, pain scores and range of motion were analyzed at 3, 6 and 12 months. Statistical analysis was performed with factors for treatment and age groups, Neer fracture types and follow-up periods, and with the consideration of age as a covariate. *Results*: DASH and CS improved following cement augmentation at three and six months compared to bone grafting, being significant when correcting for age as a covariate (*p* ≤ 0.007). While the age group had a significant effect on both these scores with worsened values at a higher age for non-augmented and grafted patients (*p* ≤ 0.044), this was not the case for cement augmented patients (*p* ≥ 0.128). Cement augmentation demonstrated good clinical results at 12 months with a mean DASH of 10.21 and mean CS percentage of 84.83% versus the contralateral side, not being significantly different among the techniques (*p* ≥ 0.372), despite the cement augmented group representing the older population with more four-part fractures. There were no concerning adverse events specifically related to the novel technique. *Conclusions*: This study has detailed a novel technique for the treatment of metaphyseal defects with PMMA cement augmentation in elderly patients with complex proximal humerus fractures and follow-up to one year, whereby the cement was allowed to partially cure to achieve medium viscosity, and then it was manually placed into the defect through the traumatic lateral window. The results demonstrate clinically equivalent short-term results to 6 months compared to augmentation with bone graft or no augmentation—despite the patient group being older and with a higher rate of more severe fracture patterns. The technique appears to be safe with no specifically related adverse events and can be added in the surgeon’s armamentarium for the treatment of these difficult to manage fractures.

## 1. Introduction

The operative treatment of proximal humerus fractures in the elderly remains problematic, with high complication rates and overall incidence of adverse events ranging from 33–48% [1,2,3]. Over half of them are implant associated and mostly result from screw cut-out or penetration [2,4]. Implant failure and varus collapse are thought to be related to avascular necrosis (AVN) and non-union. The advent of locked plating has certainly improved results, however, the use of rigid constructs with poor purchase in osteoporotic bone can lead to fragment settling and en-bloc back-out. Therefore, total shoulder arthroplasty is a resque treatment option, especially for complex fractures of osteoporotic patients. However, joint preserving treatment with locking plates remains the most often applied surgical treatment [5,6]. Augmentation of osteosynthesis with polymethylmethacrylate (PMMA) and organic bone cements has been investigated to improve fixation strength, mostly focused on screw tips with volumes of 0.5–1.0 mL per screw. Biomechanical studies have demonstrated overwhelmingly positive results, increasing load and cycles to failure in both proximal femur and humerus [7,8]. The clinical improvement has been harder to quantify with case series demonstrating reasonable results, however, the only multicenter trial for proximal humerus fractures did not demonstrate significant improvements in failure rate or outcomes [7,9].

Metaphyseal voids in the humeral head commonly occur after impaction of the shaft into the soft trabecular bone, especially in varus displacement. The fixed-angle plate construct has been proposed to act as a tension band, pulling the head out of the varus and opposing the forces of the rotator cuff. A metaphyseal defect may provide a mechanical disadvantage in the absence of an internal buttress preventing compressive collapse. In such cases, the resistance against the varus displacement of the screw-plate construct relies on the pull-out strength of the screws only and the effective length of the screws is shortened [10,11]. Finite element analysis has recommended that cement augmentation is most effective in areas of the poorest bone quality [12]. Studies on the bone density of the proximal humerus in the case of osteoporosis demonstrate the highest density at the subchondral bone (the location of screw tips) with a disproportionate decrease in the metaphyseal regions, which may not withstand compressive forces in the setting of a rigid plate construct [13]. 

Cement augmentation of metaphyseal voids has been proposed as a mechanism to improve construct stability and decrease failure rates by acting as an internal buttress. The prior literature focuses on the use of organic calcium sulphate or calcium phosphate cements with biomechanical and clinical evidence of improvement. Kwon et al. simulated the impaction in the inferior humeral head and demonstrated an improvement in the torque to failure and decrease in interfragmentary motions following void augmentation with the calcium phosphate cement using various fixation methods [14]. Kennedy et al. packed calcium triphosphate into a bone defect representing 20% of the head volume in 14 cadaveric shoulders and reported improved construct stability and load to failure [10]. Clinical case series of 22 and 29 proximal humerus fractures treated with calcium sulphate and calcium phosphate cements reported good clinical results in terms of complication profile, union rates and functional outcomes [15,16]. Calcium sulphate and calcium phosphate are both osteoconductive and can theoretically undergo resorption and replacement with new bone [17]. The latter is thought to resorb over months to years, compared with weeks to months for the former being criticized for issues with excess early wound drainage [17]. A retrospective review of patients with metaphyseal defects treated with no augmentation, cancellous bone chips or calcium phosphate cement, assessing humeral head height, neck-shaft angle (NSA) and screw-tip distance to the joint on postoperative anteroposterior radiographs, reported significantly decreased humeral head settling and joint penetration—based on these radiological markers—with use of calcium phosphate cement injected into the defect [18]. 

Calcium phosphate cement has been favored to fill bony defects in the operative treatment of younger patients, where resorption and replacement with new bone is more likely and more important. The use of PMMA-based cements, providing higher stiffness and increased compressive strength compared to calcium phosphate cements, has been the preference for the treatment of elderly patients with osteoporotic fractures [19]. PMMA is biocompatible but inert, and will not undergo remodeling, which is a potential disadvantage. The remodeling potential of calcium phosphate for void filling in elderly patients is questionable—a study reported variable and unpredictable resorption, with no patients having a complete resorption at a 10-year review [20]. Calcium phosphate cements can also demonstrate brittle fracture behavior [19]. The disadvantages of PMMA use include higher polymerization temperature, which could theoretically lead to local tissue necrosis, impairing vascularity or bone union. Interposition of an inert cement between fracture fragments would naturally impede fracture healing. Nevertheless, no thermal adverse effects of larger 10 mL PMMA volumes in osteoporotic bone have reported middle to long term for vertebroplasty in vivo [21,22]. It is likely that blood flow and heat transfer are protective for tissues that can undergo local regeneration [23]. The improved compressive strength and modulus of elasticity may be beneficial in the proximal humerus, where PMMA augmentation could provide both a compressive buttress and support for rotational movements. The other advantages of PMMA include global availability, familiarity to orthopedic surgeons, proven biomechanical properties and cost-effectiveness [23]. A single case report details the use of PMMA metaphyseal void augmentation in a revision case with a bony defect after failure due to a fall on day four post operation, reporting an uneventful union without side effects [24]. 

The first author’s practice has been offering elderly patients with complex three- or four-part proximal humerus fractures and metaphyseal voids a novel technique for augmentation with PMMA bone cement, whereby the latter was allowed to partially cure after mixing to achieve medium viscosity and then it was manually placed into the defect through the traumatic lateral window. The aim of this study was to assess the clinical outcomes and complication profiles of the novel technique for PMMA augmentation, compared to the iliac crest bone graft augmentation or no augmentation.

## 2. Materials and Methods

This Level 2 retrospective cohort study was conducted in line with the principles of the Declaration of Helsinki. Approval was granted by the local Ethics Committee (NTTMV-16970/2021).

### 2.1. Pre-Operative

This investigation included 120 patients with a proximal humerus fracture occurring in the period 2018–2021, presented to a Level 1 trauma center and treated by a single surgeon via open reduction and internal fixation. Informed consent was obtained from all participants. Following a pre-operative assessment, each patient was offered treatment with either no augmentation, or if indicated, a metaphyseal void was filled with an autologous bone graft or PMMA-based bone cement.

### 2.2. Operative

The operative approach was deltopectoral or deltoid-split, depending on the fracture characteristics. Autologous bone graft was harvested from the iliac crest in a standard manner. Fractures were fixed with a PHILOS plate (DePuy Synthes, Zuchwil, Switzerland), using at least four locking screws in the humeral head with lengths assessed per the Spross protocol [25]. 

### 2.3. Novel Technique of PMMA Metaphyseal Void Augmentation

**Figure 1:**
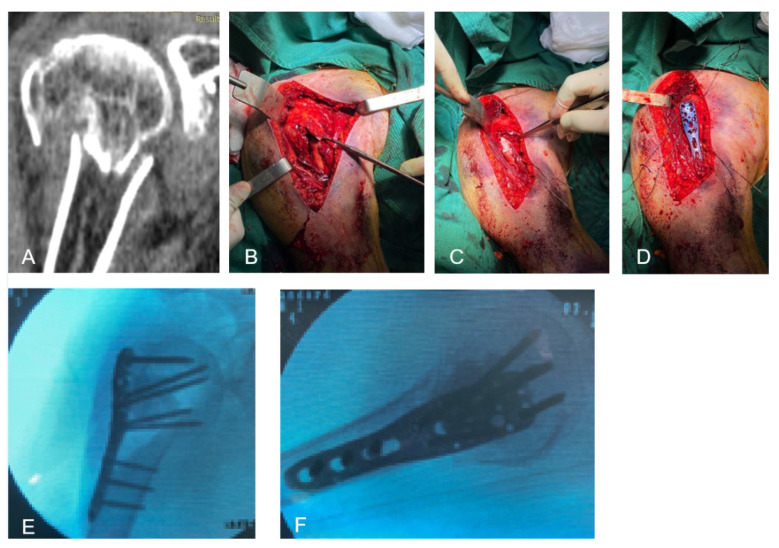
(**A**) Preoperative computed tomography scan with bone impaction and metaphyseal void; (**B**,**C**) intraoperative images with metaphyseal void and PMMA cement placement through the lateral window; (**D**) plate positioning; (**E**,**F**) anteroposterior and lateral intraoperative X-ray images.

PMMA-based bone cement (TRAUMACEM, DePuy Synthes, Raynham, MA, USA) was prepared according to the manufacturer’s instructions and allowed to partially cure for five to seven minutes after mixing to achieve medium viscosity; then, it was manually placed into the defect through the traumatic lateral window with a volume of 4–6 mL per patient, taking care to avoid any interposition between fracture fragments or any cement leakage medially (Figure 1). If the void was in the path of the cranial locking screws, the screw holes were drilled through bone and cement before its hardening. Standard wound closure was performed. 

### 2.4. Post-Operative

All patients were placed in a sling and passive assisted range of motion (ROM) was allowed between 3–6 weeks postoperatively (post-op), with gradually increasing active movements at 6–8 weeks. A postoperative pain survey was conducted at 3, 6 and 12 months, asking whether the patients had no, little, moderate or severe pain.

Radiological review was performed at 3, 6 and 12 months post-op. ROM was assessed in terms of internal and external rotation, flexion and abduction. Patient functional outcome scores of inverted Disabilities of the Arm, Shoulder and Hand (DASH) and Constant & Murley (CS) were recorded. Any operative or postoperative complications were recorded. 

### 2.5. Analysis

Fracture types were classified according to the Neer system. A postoperative assessment of reduction quality was made according to the method of Schnetzke—based on the three parameters head-shaft displacement (HSD), NSA and greater tuberosity (GT) cranialization (Table 1) [26]. Using the scoring of these parameters, cases were assigned as being with either (1) anatomical reduction if all parameters were anatomical, (2) acceptable reduction or (3) malreduction if any of the parameters were classed as malreduced. Outcomes in terms of functional scores and ROM were compared at each follow-up. Furthermore, the functional scores were investigated across categorical age groups below 60, 60–80 or over 80 years, and also with consideration of the age as a covariate. 

Statistical analysis was performed using SPSS software package (v.27, IBM SPSS, NY, USA). Normality of data distribution was screened and proved with the Shapiro-Wilk test. One-Way Analysis of Variance (ANOVA), General Linear Model, Independent- and Paired-Samples *t*-tests, and Chi-Square tests were applied to detect significant differences in outcomes among treatment groups, Neer fracture types, age groups, at follow-ups, and considering the patient characteristics. Level of significance was set to 0.05 for all statistical tests.

## 3. Results

This study assessed 63 cases with no augmentation, 28 cases with autologous bone graft augmentation and 29 cases with PMMA augmentation for a total of 120 patients. There was some heterogeneity between treatment groups (Table 2), with the PMMA augmentation group being significantly older than the other two groups (*p* ≤ 0.024), the other groups not being significantly different (*p* = 0.905). Moreover, both bone graft augmentation and PMMA augmentation groups had a significantly higher proportion of four-part fractures than the non-augmentation group (*p* ≤ 0.002) without a significant difference between the augmentation groups (*p* = 0.508). The quality of fracture reduction was not significantly different across treatment groups post operation and at 12 months (*p* ≥ 0.172). DASH and CS scores were not significantly different across treatment groups at each separate follow-up (Table 3 and Table 4, *p* ≥ 0.102). For each separate follow-up, the age group had a significant effect on both DASH and CS scores in both non-augmentation and bone graft augmentation groups (*p* ≤ 0.044), but not in the PMMA augmentation group (*p* ≥ 0.128). 

Neer fracture type did not have a significant influence on both DASH and CS (*p* ≥ 0.153). Considering age as a covariate, the PMMA augmentation group had significantly better DASH and CS scores compared to both non-augmentation and bone graft augmentation groups at all follow-ups except for DASH at 12 months (*p* ≤ 0.040 and *p* = 0.288, respectively). In contrast, DASH and CS in the non-augmentation and graft augmentation groups were not significantly different at each separate follow-up (*p* ≥ 0.167). There was no significant difference between the groups in terms of CS difference to the contralateral side at 12 months (*p* = 0.381). 

The assignment of CS at 12 months follow-up in categories of poor (≤50%), satisfactory (51–70%), good (71–90%) and excellent (>90%) outcomes revealed no significant differences in the distribution of each category among the groups (*p* = 0.365). Both DASH and CS improved significantly at each subsequent follow-up for every separate group (*p* < 0.001). 

**Figure 2:**
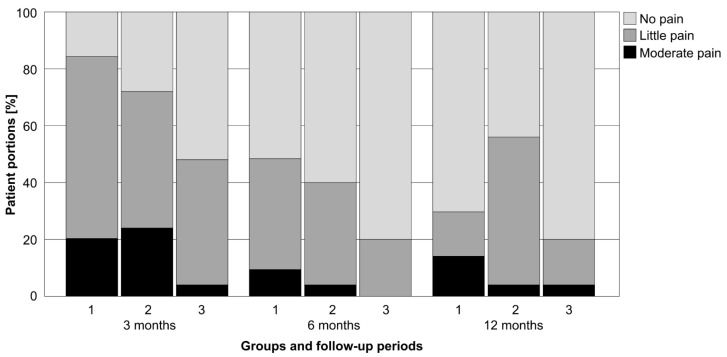
Normalized distribution of patient proportions across treatment groups (1—non-augmentation, 2—bone graft augmentation, 3—PMMA augmentation) and follow-up periods (3, 6 and 12 months) regarding self-reported pain levels for no, little or moderate pain.

There were significant differences in the distribution of patient self-reported pain levels for no, little and moderate pain across treatment groups and follow-ups (*p* ≤ 0.042). No severe pain was reported at any time. The PMMA augmentation group had a significantly higher proportion of patients reporting no pain versus the non-augmentation group at 3 and 6 months (*p* = 0.044), and with a significantly lower proportion of patients reporting moderate pain versus both non-augmentation and bone graft augmentation groups at 3 months (*p* ≤ 0.021, Figure 2). Moreover, the bone graft augmentation group had a significantly higher proportion of patients reporting moderate pain versus both the non-augmentation and PMMA augmentation groups at 12 months (*p* ≤ 0.025), the latter two being non-significantly different with regard to the self-reported pain levels (*p* = 0.396). No further significant differences were detected among pain levels, treatment groups and follow-ups (*p* ≥ 0.167).

Patient’s ROM in terms of internal and external rotation, flexion and abduction were not significantly different across treatment groups for each separate follow-up (*p* ≥ 0.154) and increased significantly at each subsequent follow-up for every separate group (*p* < 0.001, Table 5). Neer fracture type did not significantly influence ROM in each separate group and follow-up (*p* ≥ 0.125), whereas higher-age groups were significantly associated with decreased ROM in the non-augmentation and bone graft augmentation groups (*p* ≤ 0.047), but not in the PMMA augmentation group (*p* ≥ 0.151).

No significant differences were detected in the number of complication cases among the treatment groups (*p* = 0.118, Table 6). Important complications included a single example of drill bit breakage in the PMMA augmentation group, thought to result from the divergent angulation of the drill bit through the PMMA due to surgical error, which could happen in the standard procedure through a plate hole. No instances of secondary screw perforation were observed in the PMMA augmentation group, in contrast to the other two groups. Two cases of GT resorption occurred in the PMMA augmentation group. There were seven reoperations overall (5.9%)—two in the bone graft augmentation group and five in the non-augmentation group—with six patients having removal of the metal hardware and acromioplasty, and one undergoing reverse total shoulder arthroplasty due to loss of position, secondary screw cut-out and AVN. 

## 4. Discussion

This clinical study demonstrated similar results in the outcome scores, ROM and complication profiles for patients treated with no augmentation and patients with metaphyseal voids treated with either autologous bone graft or the novel technique of PMMA cement augmentation. This is despite the important variation in clinical indications leading to heterogeneous groups, with PMMA augmentation patients being older and with a higher incidence of Neer type 4 fracture patterns—which are factors that have been demonstrated and could theoretically be associated with inferior clinical results [27]. The PMMA augmentation group was actually with improved DASH and CS scores at 3 and 6 months compared to the bone graft augmentation group, which were significant when correcting for age as a covariate. Furthermore, the age categorization of patients had significant worsened functional scores at higher age groups for non-augmented and grafted patients, but not for PMMA augmented cases. At the 12 months follow-up, the results were similar. 

While the CS combines patient self-reported outcomes with a clinical assessment of ROM and strength, the DASH score is solely a patient-reported outcome measure. CS has been criticized because its values decrease with age; thus, the percentage difference compared to the contralateral side has been proposed as the most valid marker of functional outcomes [28,29]. This study demonstrated no significant differences in the percentage of CS compared to the contralateral side at 12 months follow-up. 

Only patients with more severe three- and four-part proximal humerus fractures were included in this investigation, having been associated with higher complication rates than simple fracture patterns. There was no association between patients with three- or four-part fractures and clinical outcome scores or ROM, which finding is consistent with previous reports [11,30]. The quality of reduction has been linked to higher outcome scores after three- and four-part proximal humerus fractures [26]. It is hard to compare across diverse study populations, however, the patient outcomes in this study with CS above 75 and DASH around 10 are comparable or better than those reported in other studies [4,29]. 

The complication rates in the current investigation were also comparable to previous work. A total of 63 complications occurred for 120 procedures with 7 reoperations (5.8%). A systematic review of 12 studies and a cumulative one including 719 patients reported rates of AVN 7.9%, cut-out 11.6% and reoperation 13.7% [31]. A separate meta-analysis reported an overall complication rate of 48.8% and reoperation rate of 13.8% with high rates of varus malunion (16.3%), AVN (10.8%), screw perforation into the joint (7.5%), subacromial impingement (4.8%) and infection (3.5%) [4]. The high rates of implant failure related to the collapse and screw cut-out are the very reason why novel approaches to augmentation in the fixation of proximal humerus fractures are indicated. Complication rates have decreased with the uptake of reverse total shoulder arthroplasty in displaced four-part fractures in elderly patients, and this should be considered as a treatment option in such patients [32].

There were no obviously concerning complications related to the use of PMMA for filling metaphyseal voids. No signs of bone resorption were observed specifically around the void in postoperative radiographs. The two cases of GT resorption warrant careful follow-up and are not necessarily related to the PMMA augmentation but rather to the higher age and more severe fracture patterns observed in this group. A retrospective analysis in 2021 reported GT resorption correlating with malreduction, inadequate medial support, comminution and bone quality, though the authors did not specifically look into cement augmentation [33]. The fact that there were no cases of secondary screw perforation following the PMMA augmentation may suggest improved biomechanical stability. The potential for heat-related damage and necrosis needs to be kept into consideration. Osteocyte thermal necrosis is related to time and temperature of exposure, with apoptosis thought to occur with exposure at threshold values of 50.8 °C for one minute or 48.8 °C for ten minutes, and partial necrosis if the exposure is at a threshold value of 47.8 °C for five minutes or between 42–45 °C for ten minutes [34]. The temperature increase in vivo is related to the specific polymerization reaction and the cement volume. Small volumes of 0.5–1.0 mL at the screw tips appear safe—a recent study injecting larger volumes to replace screws rather than augment screw tips reported temperatures that would be associated with partial cell death but not with complete necrosis [34,35]. In this study, volumes of 4–6 mL per patient were utilized, being smaller than volumes used clinically for vertebroplasty without significant long-term complications [21,22].

The augmentation of proximal humerus fractures in the biomechanics laboratory with PMMA or calcium phosphate cement has consistently demonstrated improved stability—augmenting screw tips with PMMA and filling metaphyseal voids with calcium phosphate demonstrated increased load and cycles to failure, and reduced interfragmentary motions [7,8,10,36,37]. Further biomechanical investigations comparing the stability of metaphyseal augmentation with calcium phosphate and PMMA-based cements is warranted to determine whether the higher compressive strength and Young’s modulus of PMMA translates to a potentially clinically significant improvement in construct stability. PMMA-tricalcium phosphate composites may provide greater mechanical strength with some biological activity. Despite a mountain of biomechanical evidence, the clinical benefit to PMMA screw tip augmentation remains to be conclusively demonstrated—the only randomized multicenter trial reported no significant differences between groups in terms of function and adverse events; however, it was underpowered and with overall complication rate in both groups being lower than anticipated [9]. Further prospective randomized trials and biomechanical studies comparing augmentation methods in proximal humerus fractures are indicated to investigate optimal treatment methods in a procedure with high failure rates. The differences in the distribution of patient self-reported pain scores among the treatment groups are of unclear clinical significance and may represent age-related differences in pain tolerance. The clinically important aspect is that there was no obvious increase in the patient-reported postoperative pain that might indicate local bone or tissue damage from thermal necrosis.

The limitations of this study include the lack of strict patient randomization resulting in heterogeneous treatment groups. The authors believe that there are distinct indications for the use of different techniques in these groups, but the importance of this investigation lies in the demonstration of similar results with the application of the novel cement augmentation technique despite the older corresponding patient group. The use of the autologous bone graft may be supported in younger patients with a greater healing capacity for remodeling. The well-known issues with donor site morbidity, pain and potential infection may not be justifiable in the older population if there are no direct clinical benefits. The harvesting of autologous bone graft from the iliac crest is also more difficult in the beach chair position compared to lower limb procedures. Longer follow-up reporting of this novel technique in the future will be important. The fact that procedures and follow-up assessments were conducted by a single surgeon at a single center with a consistent technique adds to the validity of the results.

## 5. Conclusions

This study has detailed a novel technique for the treatment of metaphyseal defects with PMMA cement augmentation in elderly patients with complex proximal humerus fractures and follow-up to one year, whereby the cement was allowed to partially cure to achieve medium viscosity; then, it was manually placed into the defect through the traumatic lateral window. The results demonstrate clinically equivalent short-term results to 6 months compared to augmentation with bone graft, or no augmentation—despite the patient group being older and with a higher rate of more severe fracture patterns. The technique appears to be safe with no specifically related adverse events and can be added in the surgeon’s armamentarium for the treatment of these difficult to manage fractures. Further biomechanical and clinical trials will delineate more clearly the clinical benefits of this method.

## Figures and Tables

**Table 1:** Ranges of the three parameters for postoperative assessment of fracture reduction quality HSD, NSA and GT cranialization (GTC) according to the method of Schnetzke.

Parameter	Range of Fracture Reduction		
	Anatomical	Acceptable	Malreduced
HSD (mm)	Anatomical	≤5	>5
NSA (°)	Normal (120–150)	Minor varus (110–120)	>150 (valgus) or <110 (major varus)
GTC (mm)	Anatomical	≤5	>5

**Table 2:** Number of cases in the treatment groups according to their characteristics, together with age presented in terms of mean value and standard deviation.

Treatment Group	Number of Cases					Age (Years)
	Female	Male	Total	Neer Classification		
				Three-Part	Four-Part	
Non-augmentation	56 (89%)	7 (11%)	63	61 (97%)	2 (3%)	63.4 ± 12.7
Bone graft augmentation	25 (89%)	3 (11%)	28	24 (86%)	4 (14%)	64.5 ± 10.6
PMMA augmentation	28 (97%)	1 (3%)	29	21 (72%)	8 (28%)	71.9 ± 10.7

**Table 3:** DASH score in the treatment groups at three different follow-up periods, presented in terms of mean value and standard deviation.

Treatment Group	Follow-Up Period (Months)		
	3	6	12
Non-augmentation	24.77 ± 7.51	15.19 ± 6.93	8.42 ± 5.59
Bone graft augmentation	26.86 ± 7.12	18.29 ± 6.30	10.15 ± 5.32
PMMA augmentation	23.67 ± 7.95	15.53 ± 5.91	10.21 ± 6.83

**Table 4:** CS score in the treatment groups at three different follow-up periods, presented in terms of mean value and standard deviation.

Treatment Group	Follow-Up Period (Months)			Difference to Contralateral at 12 Months
	3	6	12	
Non-augmentation	49.03 ± 12.95	68.08 ± 12.74	80.53 ± 12.12	16.63 ± 11.99 (82.74 ± 12.34%)
Bone graft augmentation	45.32 ± 10.18	64.96 ± 11.19	77.36 ± 11.89	13.18 ± 8.54 (85.25 ± 9.41%)
PMMA augmentation	52.90 ± 9.59	69.90 ± 9.16	79.48 ± 14.02	13.54 ± 11.22 (84.83 ± 12.67%)

**Table 5:** Patients ROM scores in the treatment groups at three different follow-up periods, presented in terms of mean value and standard deviation.

Treatment Group	Follow-Up Period (Months)		
	3	6	12
Internal rotation (°)			
Non-augmentation	39.35 ± 9.30	55.97 ± 9.18	62.74 ± 9.86
Bone graft augmentation	40.71 ± 9.79	58.04 ± 7.86	63.39 ± 7.58
PMMA augmentation	42.59 ± 9.88	57.07 ± 10.48	64.48 ± 8.06
External rotation (°)			
Non-augmentation	51.94 ± 11.28	65.00 ± 6.07	67.42 ± 4.41
Bone graft augmentation	53.21 ± 9.64	65.89 ± 4.92	68.57 ± 3.56
PMMA augmentation	55.86 ± 8.77	65.17 ± 7.13	67.93 ± 4.12
Flexion (°)			
Non-augmentation	111.85 ± 22.73	142.50 ± 19.16	157.83 ± 15.06
Bone graft augmentation	106.96 ± 18.68	137.86 ± 15.48	154.82 ± 15.24
PMMA augmentation	113.28 ± 14.84	141.72 ± 19.93	154.14 ± 19.32
Abduction (°)			
Non-augmentation	93.31 ± 23.66	127.02 ± 21.53	146.21 ± 20.58
Bone graft augmentation	88.93 ± 21.27	119.11 ± 16.89	139.82 ± 17.87
PMMA augmentation	97.41 ± 15.27	123.28 ± 21.14	139.48 ± 23.58

**Table 6:** Intraoperative and postoperative number of complication cases in treatment groups 1 (non-augmentation), 2 (bone graft augmentation) and 3 (PMMA augmentation).

Complications (Overall Incidence)	Treatment Groups		
	1	2	3
Intraoperative			
Iatrogenic screw penetration (4.2%)	2	1	2
Intraosseous broken drill bit (0.8%)	0	0	1
Neuropraxia—lateral femoral cutaneous nerve (0.8%)	0	1	0
Postoperative			
Varus with NSA < 110° or NSA change > 10° (7.6%)	7	2	0
GT proximalization (5.0%)	3	2	1
Partial GT resorption (1.7%)	0	0	2
Subacromial impingement (6.7%)	5	2	1
Secondary screw perforation (4.2%)	3	2	0
Adhesive capsulitise (6.7%)	6	2	0
Pain at the donor site—iliac crest (1.7%)	0	2	0
AVN (6.7%)	3	3	2
Infection (1.7%)	1	1	0
Other implant-related complications (5.0%)	4	2	0

## Data Availability

The datasets analyzed during the current study are available from the corresponding author on reasonable request. In order to comply with the requirements of the Ethics Committee, the image set is not available for request due to data privacy policies.
